
JOURNAL INFORMATION
==============================
NLM Title Abbreviation: Northwest Med Surg J
NLM Journal ID: 0020727

The North-Western Medical and Surgical Journal

Publisher: Biomedical Journal Digitization Project

ARTICLE INFORMATION
==============================
PMCID: PMC9973789
PMID: 37320727
Article version: 1
Subjects: Editorial

Professional Changes

Print publication date: 1857 Aug
Volume: 6
Volume Series: New Ser
Issue: 8
First page: 388
Last page: 388
License: This work is free of known copyright restrictions. For more information, please see PMC Back Issue Digitization.
License URL: https://creativecommons.org/publicdomain/mark/1.0/

==============================

*Professional Changes.*

Dr. S. G. Armour, who recently resigned the Chair of Pathology and Clinical Medicine in the Ohio Medical College, at Cincinnati, has been elected to the same chair in the Missouri Medical College, of St. Louis. We are informed that Dr. Armour will make St. Louis his future place of residence.

---

**SARGENT & ILSLEY'S**

SOLUTION OF

### CHLORIDE OF ZINC;

*For Purifying Sick Chambers, Sinks, Chamber Vessels, Vaults, Cellars, and for Preserving Corpses, Preventing Moths, Exterminating Bugs, etc.*

To obtain a substance harmless in itself, and free from smell, but possessed of the property of destroying all other smells, particularly such as are offensive or injurious to health, has long occupied the attention of scientific men. It has been found that Chloride of Zinc possesses this power in a high degree, and is also safe, economical and convenient.

It has received the sanction of the highest medical authority, and been very extensively introduced into the hospitals and public institutions of Europe and this country.

Our solution is of uniform strength, containing 35 per cent. of the dry chloride, and for most purposes should be diluted with twenty times its bulk of water. It is the cheapest, most effectual and convenient disinfectant known.

Its application is perfectly safe, both to persons and property; it is also free from the noxious and disagreeable odor of the chloride of lime, and the objections pertaining to the various disinfectants in common use. Full directions accompany each bottle. Prepared by

SARGENT & ILSLEY,

Druggists, 140 Lake street, Chicago.
